# Acute infectious thyroiditis in ectopic lingual thyroid causing dysphagia and dyspnoea: a case report and discussion

**DOI:** 10.1259/bjrcr.20160025

**Published:** 2016-09-07

**Authors:** Marika Kalnina, Anda Pramalte, Liene Zemniece, Yegor Safronov

**Affiliations:** ^1^Nuclear Medicine Department, Nuclear Medicine Centre, Ltd, Riga, Latvia; ^2^Institute of Diagnostic Radiology, Pauls Stradins Clinical University Hospital, Riga, Latvia; ^3^Institute of Diagnostic Radiology, Pauls Stradins Clinical University Hospital, Riga, Latvia; ^4^Department of Otorhinolaryngology, Pauls Stradins Clinical University Hospital, Riga, Latvia

## Abstract

Acute infectious thyroiditis in patients with ectopic lingual thyroid gland is extremely rare, and in most cases, this condition, if symptomatic, manifests as dysphagia and dyspnoea. This case report shows that, in addition to the previously mentioned typical symptoms, our patient also suffered from febrile temperature, progressive fullness in the throat and pain during swallowing. Diagnosis of acute infectious thyroiditis associated with lingual thyroid gland is complex and requires evaluation of results of clinical findings and laboratory tests, and also evaluation by radiological diagnostic methods. Treatment was performed by administering antibacterial and anti-inflammatory therapy, resulting in the disappearance of complaints. Clinical appearance, diagnostic methods and treatment options are discussed in the discussion section.

## Clinical presentation

A 27-year-old female patient was admitted to the emergency room at 4 am complaining of progressive fullness in the throat, pain during swallowing, dysphagia and dyspnoea. The patient had a 3-day history of febrile temperature and her complaints had rapidly progressed in the 3-day period. After clinical investigation, the otorhinolaryngologist suspected an epiglottic abscess, and less likely an ectopic thyroid gland, as they are rare.

## Differential diagnosis

In this case, after clinical and first radiological imaging, three differential diagnoses were assumed—epiglottic abscess, thyroglossal duct cysts and a dermoid cyst.^1^ In order to assess the possibility of an epiglottic abscess, a CT scan was performed. To establish or exclude the possibility that the formation at the root of the tongue was an ectopic thyroid gland, a single photon emission CT/CT (SPECT-CT) with technetium-99m (^99m^Tc) pertechnetate was performed.

## Investigations/Imaging findings

**Figure 1.**
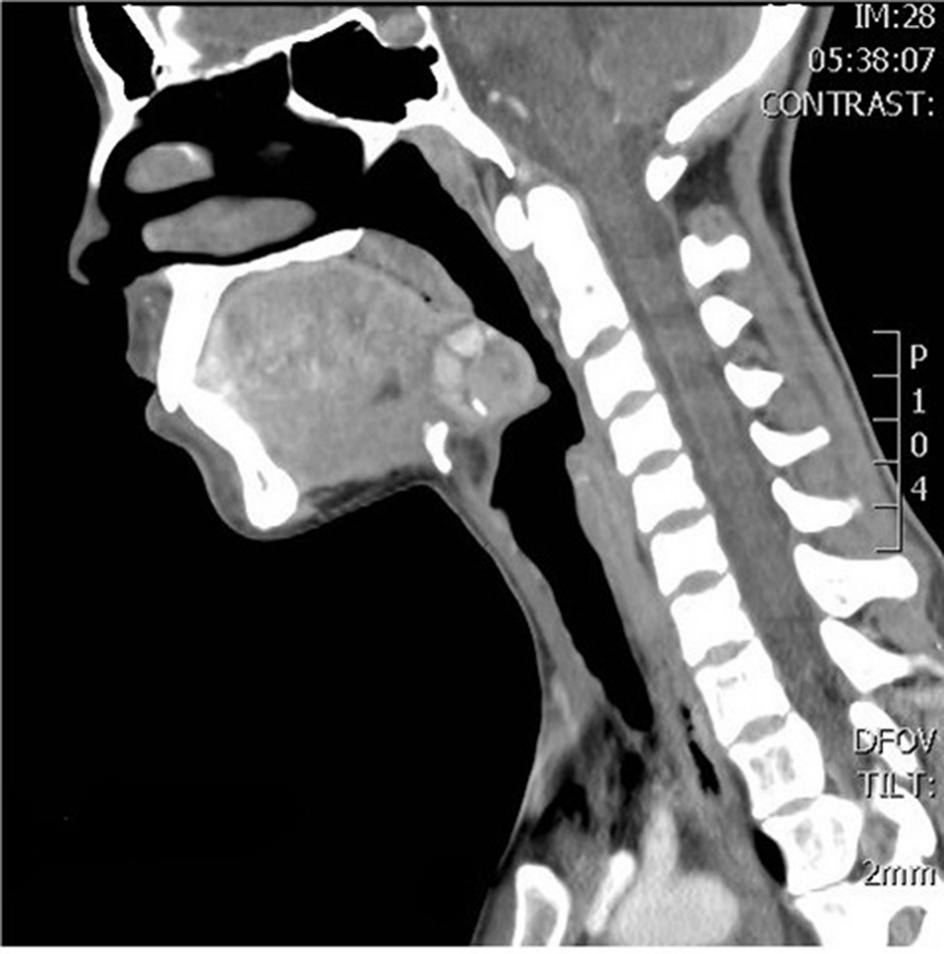
CT scan with contrast shows a non-homogeneous contrast-enhancing area (1.7cm diameter) at the root of the tongue with calcifications in the periphery. Around this structure, a few nodular structures (1cm diameter) are located, with contrast-enhancing features similar to the thyroid gland. Also, the thyroid gland cannot be visualized in its typical position in the neck. This tissue mass compresses the epiglottis and narrows the pharynx.

**Figure 2.**
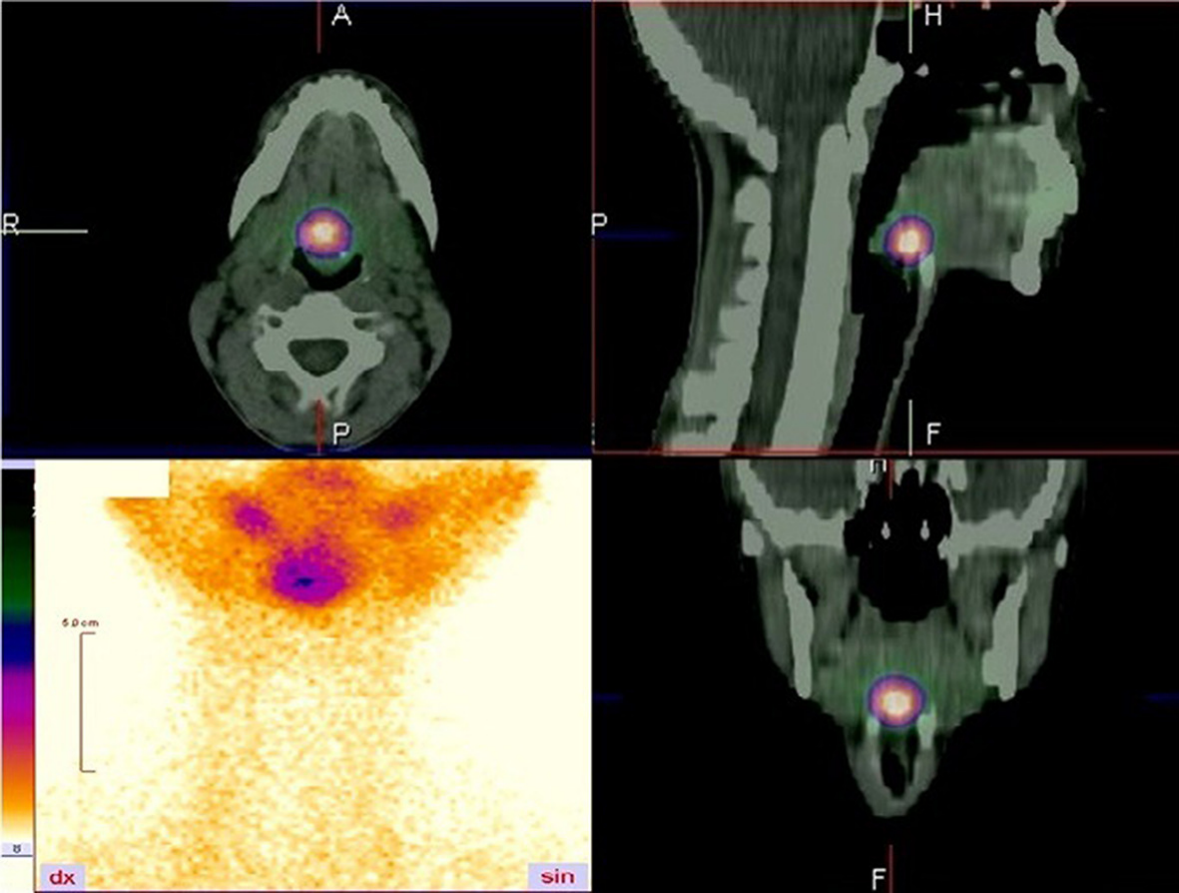
Single photon emission CT/CT using technetium-99m showed radioisotope uptake at the root of the tongue (in the same location as the mass seen on CT scan) and no uptake of radioisotope in the normal thyroid location.

Blood tests revealed leukocytosis, “left shift” and elevated C-reactive protein. Thyroid-stimulating hormones, triiodothyronine and thyroxine levels were within the normal range. Laryngoscopy revealed a non-homogeneous mass-like prominence at the root of the tongue. Visual examination showed no convincing signs of inflammation in the mucosa of the throat, only local redness at the root of the tongue. The patient was first examined with ultrasound imaging, but in standard localization, the thyroid gland was not found; therefore, a contrast-enhanced CT scan was performed. It revealed a non-homogeneous contrast-enhancing area (1.7 cm diameter) at the root of the tongue with calcifications along the periphery. A few nodular structures (1cm diameter) were located around this structure, which enhanced the contrast similar to a thyroid gland. Also, the thyroid gland could not be visualized in its typical position in the neck. This tissue mass was shown to be compressing the epiglottis and narrowing the pharynx (Figure 1). SPECT-CT using ^99m^Tc pertechnetate showed a homogeneous radioisotope uptake at the root of the tongue (corresponding to the mass seen on the CT scan) and no uptake of the radioisotope at the normal location of the thyroid gland (Figure 2). SPECT-CT scan was performed 2 weeks later, when her complaints dissipated, to confirm the diagnosis of an ectopic thyroid gland.

## Treatment

The patient received antibiotic and anti-inflammatory therapy, and her complaints disappeared within 4 days.

## Outcome, follow-up and discussion

After starting antibiotic and anti-inflammatory therapy, the patient's complaints disappeared within 4 days. After 1 month, a follow-up laryngoscopy showed that the ectopic lingual thyroid gland had decreased in size. Examination after 2 months showed that the ectopic thyroid gland was continuing to decrease in size. The patient did not have any complaints of fullness in the throat, dysphagia or dyspnoea. Blood investigations and biochemical analysis, including thyroid-stimulating hormones, triiodothyronine and thyroxine, were within normal range. An ectopic thyroid gland is a functioning gland, which is located away from its usual position in the lower neck. An ectopic thyroid gland develops as a result of abnormal embryological migration from the foramen caecum to the usual location of the gland in the pretracheal area. An ectopic thyroid gland can develop anywhere in the path of migration of the gland from the foramen caecum to its usual location.^1,2^ When migration of the thyroid gland stops at the base of the tongue, it results in a lingual thyroid. The incidence of ectopic thyroid gland is unknown because, in many cases, the thyroid tissue is asymptomatic and symptoms occur only if the ectopic thyroid gland becomes enlarged. Enlargement of the thyroid gland can occur at all ages, but it occurs most often during puberty, gestation or while experiencing upper respiratory tract infections.^1,3^ If symptomatic, the clinical presentation may vary, depending on the location of the thyroid gland. A lingual thyroid gland can manifest as dysphagia, dyspnoea or dysphonia.^1^ Acute infectious thyroiditis related to lingual thyroid gland is rare,^4^ and in the medical literature, it has been described very sparingly. The typical clinical symptoms of acute infectious thyroiditis are febrile temperature and painful thyroid gland with local redness. Diagnosing an ectopic thyroid gland is not problematic if the clinician has considered the possibility of thyroid ectopia, because radiological examinations and nuclear medicine tests, such as scintigraphy, are sufficiently informative.^5,6^ Ultrasound, CT scans and MRI help in visualizing the atypical anatomical structures at the root of the lingua. Thyroid gland scintigraphy with ^99m^Tc pertechnetate reveals radioisotope uptake by functional thyroid tissues located away from the typical position of the gland in the neck, and summing up the results of the radiological and functional nuclear medicine tests can provide a true diagnosis of an ectopic thyroid gland. Once the diagnosis is confirmed, treatment is required. A few treatment points are described later in this report. Treatment of this condition depends on results of laboratory and functional test, and radiological imaging. If the patient has no symptoms, observation is often required, especially in cases where the thyroid gland is functioning well.^7^ In many cases, ectopia of the thyroid gland is associated with hypothyroidism. In approximately one-third of cases, there is decrease in functioning of the thyroid gland,^5^ leading to the use of thyroid hormone.^1^ If the thyroid gland is enlarged, homone suppression therapy may be necessary to minimize the size of the gland; radioiodine ablation may be used in patients with progressive and symptomatic enlargement of the thyroid gland.^2^ Surgical treatment (thyroidectomy) is not necessary in most cases, but surgery can be performed if the symptoms progress despite medical management. Surgery is also required if the ectopic thyroid gland is associated with malignant thyroid disease.^1,5^ Carcinoma arising in a lingual thyroid gland is extremely rare, with an estimated incidence of 1%, often occurring during the third decade of life.^8^ In our case, the patient had symptoms typical of lingual thyroid gland, such as fullness in the throat, dysphagia and dyspnoea, but, in addition to these symptoms, she also had febrile temperature and pain during swallowing, which is not typical of an ectopic thyroid gland; therefore, the differential diagnosis of abscess was considered. In this case, the patient had an ultrasound performed, but the thyroid gland was not located in its usual position in the neck; therefore, a contrast-enhanced CT scan was required, which showed a structure at the root of the tongue with other suspected diagnoses. After ^99m^Tc pertechnetate scintigraphy of the thyroid gland was performed, it became clear that the lingual structure was an ectopic thyroid lesion, and according to laboratory findings and clinical investigation, it seemed to be acute infectious thyroiditis associated with lingual thyroid gland. It was assumed that the lingual thyroid gland had increased in size during the phase of acute infectious thyroiditis. This is rare; therefore, the patient was treated with antibiotics and anti-inflammatory medication, and the treatment was successful—symptoms diminished and blood tests improved. Surgical treatment was not considered as there was no suspicion of malignancy.

## Learning points

1. Ectopic thyroid gland tissue is a rare entity that results from developmental defects during the early stages of thyroid gland embryogenesis.
2. Enlargement of the thyroid gland can occur most commonly during pregnancy or while suffering from upper respiratory tract infections,^1^ but it is rarely associated with acute infectious thyroiditis.
3. The diagnosis of lingual thyroid gland depends on the awareness of the clinician,^5^ because targeted examinations of the suspected ectopic thyroid gland easily lead to the right diagnosis. The diagnosis of acute infectious thyroiditis associated with lingual thyroid gland is complex and requires evaluation of the clinical findings, laboratory results and radiological diagnostic methods.
4. Thyroid gland scintigraphy with ^99m^Tc pertechnetate is the most precise method for diagnosing an ectopic thyroid gland.
5. In cases of acute infectious thyroiditis, antibacterial treatment is used. To minimize the size of the symptomatic lingual thyroid gland, hormone suppression therapy, or radioiodine ablation, may be used. In most cases, thyroidectomy is not necessary; it is only required if a high risk of malignancy is suspected or a progression in size occurs despite medical management.
6. To avoid a possible recurrence or complications, follow-up is recommended.

## Consent

Informed consent was signed by the patient and identifying information has not been used in this case report.
